# Early Treatment Initiation With Oral Prednisolone for Relapse Prevention Alleviates Depression and Fatigue in Aquaporin-4–Positive *Neuromyelitis optica* Spectrum Disorder

**DOI:** 10.3389/fneur.2021.608149

**Published:** 2021-02-22

**Authors:** Tetsuya Akaishi, Toshiyuki Takahashi, Kazuo Fujihara, Tatsuro Misu, Juichi Fujimori, Yoshiki Takai, Shuhei Nishiyama, Michiaki Abe, Tadashi Ishii, Masashi Aoki, Ichiro Nakashima

**Affiliations:** ^1^Department of Neurology, Tohoku University Graduate School of Medicine, Sendai, Japan; ^2^Department of Education and Support for Regional Medicine, Tohoku University Hospital, Sendai, Japan; ^3^Department of Neurology, National Hospital Organization Yonezawa National Hospital, Yonezawa, Japan; ^4^Department of Multiple Sclerosis Therapeutics, Fukushima Medical University School of Medicine, Fukushima, Japan; ^5^Department of Neurology, Tohoku Medical and Pharmaceutical University, Sendai, Japan

**Keywords:** anti-aquaporin-4 antibodies, depression, fatigue, neuromyelitis optica spectrum disorders, oral prednisolone, psychiatric disturbances

## Abstract

**Background:** *Neuromyelitis optica* spectrum disorder (NMOSD) is a relapsing autoimmune-related neurological disorder of the central nervous system. Most patients with NMOSD have serum anti-aquaporin-4 immunoglobulin G antibodies (AQP4-IgG). In addition to optic neuritis and myelitis, other insidious symptoms such as depressive state and chronic fatigue in NMOSD are gradually being recognized.

**Methods:** To elucidate the impact of low- to medium-dose oral prednisolone (PSL) as a relapse prevention therapy for psychiatric disturbances and chronic fatigue in NMOSD, we evaluated clinical data from 39 patients with AQP4-IgG-positive NMOSD, along with the details of present and cumulative oral PSL dosage.

**Results:** Thirty-six of the 39 patients were treated with low- to medium-dose oral PSL, and the mean and standard deviation of the present daily dose of oral PSL were 7.9 ± 4.0 mg/day. None of the patients were treated with a daily PSL dose of >15 mg. As a result, the disease duration and the untreated period before starting oral PSL showed weak to moderate correlations with the subsequent severities of psychiatric disturbance and fatigue level. Meanwhile, none of the other treatment-related variables evaluated, such as the present oral PSL daily dose, cumulative PSL dose, months of oral PSL administration, previous courses of steroid pulse therapy, and coadministered immunosuppressants, were correlated with these insidious symptoms.

**Conclusion:** Our results suggest that the use of long-term low- to medium-dose oral PSL ≤15 mg daily for relapse prevention in AQP4-IgG-positive NMOSD would not aggravate the psychiatric and fatigue conditions. On the contrary, early initiation of oral PSL for relapse prevention, together with significantly decreased relapse rate, alleviated the subsequent depressive state and fatigue from the disease.

## Introduction

*Neuromyelitis optica* spectrum disorder (NMOSD) is a relapsing autoimmune-related neurological disorder in the central nervous system. Most of the patients with NMOSD have serum anti-aquaporin-4 immunoglobulin G antibodies (AQP4-IgG) (1–3). Patients with NMOSD typically present with symptoms based on relapsing attacks with optic neuritis and myelitis. However, it has come to be known that many patients are also accompanied by several intractable insidious symptoms such as depressive states and chronic fatigue, which are not always related to the level of neurological disability (4–8). These insidious symptoms have been known to significantly impair the quality of life (QOL) of patients, but their exact causes and mechanisms have not yet been identified (4). Patients with NMOSD have been conventionally treated by oral administration of single or combined immunosuppressants, such as azathioprine, tacrolimus, mycophenolate mofetil, or prednisolone (PSL), to prevent relapses (9–12). Although long-term administration of low- to medium-dose oral PSL is not a global standard of care to prevent relapses, it is considered to be effective for suppressing relapses in NMOSD as with other preventive therapies (13, 14). Recently, in addition to these conventional oral relapse prevention therapies, several highly efficacious parenteral monoclonal antibodies, such as rituximab, eculizumab, satralizumab, and inebilizumab, have emerged as other promising therapeutic options (15–17). Relapse prevention with these preventive therapies is essential in NMOSD as the neurological disability in the disorder is known to have a sudden occurrence with stepwise development of irreversible neurological symptoms (18–20). Furthermore, relapse prevention therapy in NMOSD is usually needed to be continued for a long period of time because relapses may take place even more than several decades after the previous attack (21). As a result, the long-term tolerability of the aforementioned therapies is an important aspect to be considered when selecting the best therapeutic plan for each patient. To date, the safety and effectiveness of these therapies on the aforementioned insidious symptoms (i.e., psychiatric conditions and chronic fatigue) in NMOSD have not been studied. In particular, the long-term administration of high-dose oral PSL (typically ≥20 mg daily) has been known to cause psychiatric side effects in nearly half of the patients, although they are usually reversible (22–24). Thus, there is a need for patients and clinicians to elucidate the safety of long-term administration of low- to medium-dose oral PSL <20 mg daily from the viewpoint of psychiatric side effects. Therefore, we retrospectively studied the correlations between the history of relapse prevention therapies, mostly with oral PSL in this study, and the above-described insidious symptoms in AQP4-IgG-positive NMOSD, to identify the impact of low- to medium-dose oral PSL on the depressive state and chronic fatigue in patients with the disease.

## Methods and Materials

### Study Design

We retrospectively collected clinical information from 39 consecutive patients with AQP4-IgG-positive NMOSD who were treated at the outpatient center of Tohoku University Hospital and agreed to participate in this study. All patients who were asked to participate in this study agreed to cooperate. The positivity of serum AQP4-IgG was checked by the microscopic live cell-based assay method using human M23-AQP4-expressing HEK293 cells, as previously done (25, 26). The patients were cross-sectionally studied for the severity of depression and fatigue between June and September in a single year. Depressive state was measured using the self-reported Quick Inventory of Depressive Symptomatology (QIDS-SR), and fatigue level was measured using the 14-item Chalder Fatigue Scale (CFS). Patients took the self-report sheets to their homes and submitted the filled sheets at the next hospital visit 1–2 months later.

### Evaluated Variables

QIDS-SR is a scale for assessing the psychiatric depressive state in the last 7 days, which comprises nine self-reported questions with scores of 0–3 for each (i.e., scores of 0–27 in total). Higher scores indicated the higher likelihood of patients experiencing depression. Those with QIDS-SR scores ≥6/27 were considered to have depressive symptoms. In this study, all 39 patients answered all nine questionnaires.

CFS is a scale for assessing the chronic fatigue state, comprising 14 questions with scores of 1–4 for each (i.e., scores of 14–56 in total) (27). Higher scores indicated that the patient was more likely to experience chronic fatigue. Those with CFS scores ≥26/56 were considered to have chronic fatigue. In this study, one patient failed to respond to one of the 14 items of the CFS, and the score was not valid for the subsequent assessment. Consequently, valid CFS data were obtained from 38 of the 39 patients.

To study the possible effects of relapse prevention therapies on these insidious symptoms, we also collected the following treatment-related information at the time of assessment: duration of the oral PSL administration (in months), the present oral PSL dosage (mg/day), the cumulative total amount of previously administered oral PSL (mg), total number of previously administered steroid pulse therapy (times), and the existence of other co-administered immunosuppressants such as azathioprine or eculizumab. All these treatment-related variables were evaluated for correlation with each of the studied insidious symptoms (i.e., depressive state and fatigue). Furthermore, the following baseline data of these 39 patients were also studied: age, sex, body weight, disease duration, previous numbers of attacks, Kurtzke Expanded Disability Status Scale score, level of gait disturbance, and the number of eyes (0–2) with legal blindness (i.e., decimal corrected visual acuity <0.1). The level of gait disturbance was categorized into the following five levels: 0 (none), 1 (mild; no disturbance in daily living), 2 (moderate; can walk but having disturbance in daily living), 3 (severe; wheelchair bound), and 4 (bedridden). As several typical cerebral lesions may accompany in nearly half of the patients with NMOSD (28–30), the presence of cerebral T2/FLAIR high-intensity lesions in the brain MRI at the time of assessment was also evaluated.

### Statistical Analysis

As for the descriptive statistics of the demographic and clinical data of the enrolled patients, quantitative variables with supposedly normal distribution were described with the mean and standard deviation (SD). Quantitative variables with non-normal distribution were described with median and interquartile range (IQR; 25–75 percentiles). Spearman's correlation coefficient (Spearman's rho) was calculated between the insidious symptoms and the studied treatment-related variables. *P* < 0.05 were considered statistically significant. The statistical analyses in this study were performed using SPSS Statistics Base 22 software (IBM) and MATLAB R2015a (MathWorks).

### Ethics

This study was approved by the Institutional Review Board of the Tohoku University School of Medicine and Tohoku University Hospital. This research was conducted in accordance with the Helsinki Declaration as revised in 2013. Each patient provided written informed consent before participating in this study.

## Results

### Patient Backgrounds

Demographic and clinical features of the enrolled 39 patients are listed in Table 1. As for the distributions of the QIDS-SR and CFS, no fewer than 23 (59.0%) of the 39 patients showed more than a weak depressive state (i.e., QIDS-SR ≥ 6/27), whereas 29 (76.3%) of the 38 patients with valid CFS data showed abnormally high CFS scores (i.e., CFS ≥ 26/56). Ten of the 39 patients (25.6%) were co-administered immunosuppressants (9 with azathioprine and 1 with eculizumab) other than oral PSL. Three of the 39 patients were not treated with any relapse prevention therapies during the assessment period because they decided against using any of the treatments even with relapsing clinical courses, after being well-explained and understanding the risk of not using the therapies. The mean ± SD of the body weight was 55.6 ± 9.1 kg, and the oral PSL daily dose adjusted by body weight was 0.151 ± 0.083 mg/kg/day.

**Table 1:** Demographic and clinical features of the enrolled 39 patients with AQP4-IgG-positive NMOSD.

**Variables**	**Data in the total enrolled patients (*n* = 39)**
Male: Female	0: 39
Age at assessment^*^	51.5 ± 13.5 years
Disease duration at assessment^†^	9.0 (5.0–15.5) years
Previous times of relapses at assessment^†^	3.0 (1.0–5.0) times
EDSS score at assessment^†^	4.5 (2.5–6.0)
Number of eyes with legal blindness^†^	1.0 (0.0–2.0) eyes
PSL daily dose at assessment^*^	7.90 ± 4.01 mg/day
PSL daily dose adjusted by body weight^*^	0.151 ± 0.083 mg/kg/day
Cumulative total administered oral PSL^†^	17,155 (10,585–26,463) mg
Total cycles of previous steroid pulse therapy^†^	2.0 (1.0–3.5) cycles
Cycles of steroid pulse therapy before starting long-term oral PSL^†^	0.0 (0.0–1.5) cycles
Co-administered immunosuppressants	*n* = 10 (25.6%)
QIDS-SR score^†^	6 (4–11)
CFS score^†^	32 (27–37)

*CFS, Chalder Fatigue Scale; EDSS, Kurtzke Expanded Disability Status Scale; PSL, prednisolone; QIDS-SR, self-reported Quick Inventory of Depressive Symptomatology*.

* *Mean and standard deviation*.

† *Median and interquartile range (25–75 percentiles)*.

### Factors That Affected Depression and Fatigue

**Figure 1:**
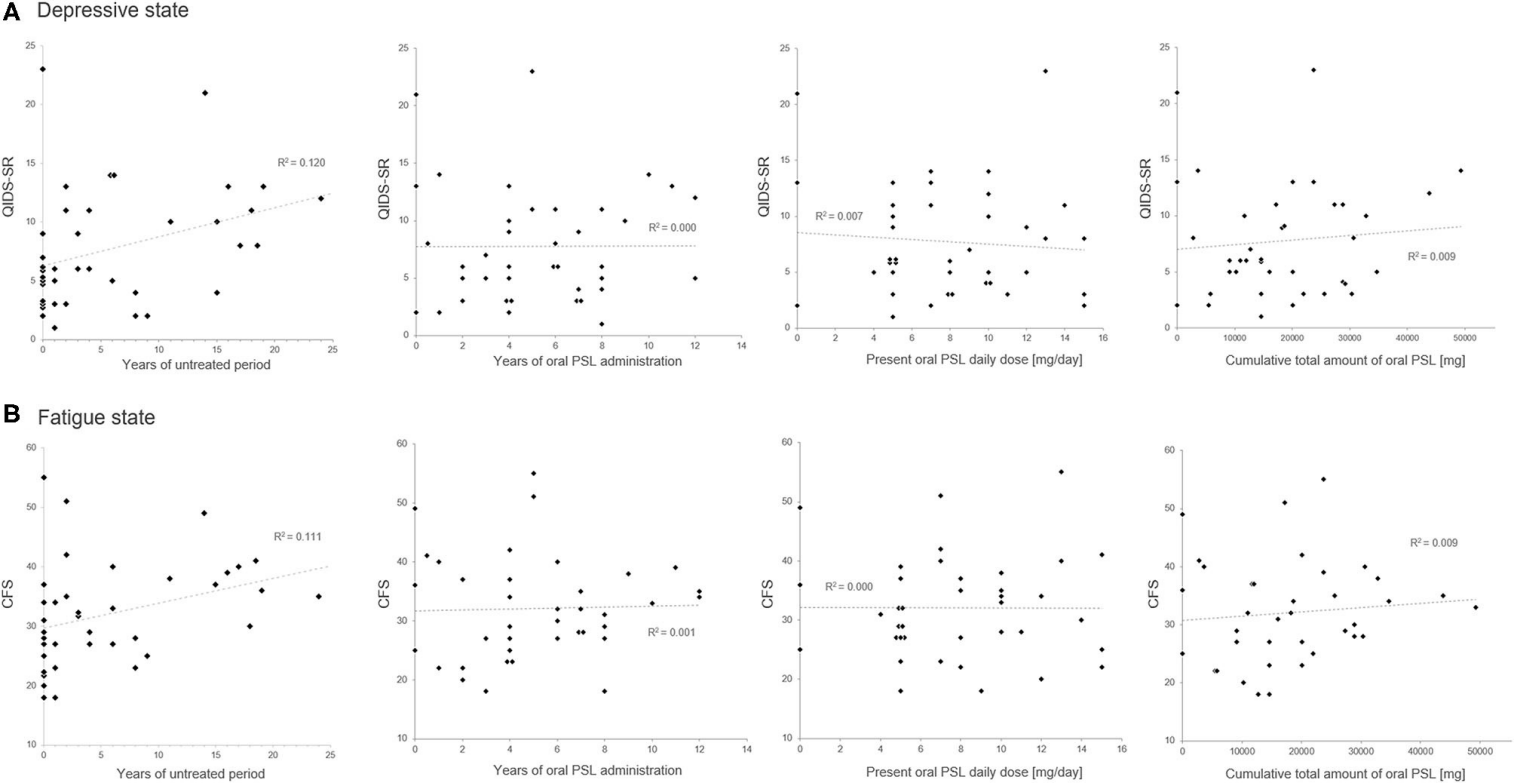
Scatter plots of the oral prednisolone-related therapeutic variables and the insidious symptoms. The duration of the untreated period shows a positive correlation with the present scores in QIDS-SR **(A)** and CFS **(B)**. Meanwhile, none of the present oral prednisolone dosage, duration of prednisolone administration, or cumulative total amount of oral prednisolone shows a significant correlation with the present depressive state or fatigue level. CFS, Chalder Fatigue Scale; PSL, prednisolone; QIDS-SR, self-reported Quick Inventory of Depressive Symptomatology.

The correlation matrix between the evaluated insidious symptoms and treatment-related variables in the enrolled 39 patients is summarized in Table 2. As shown in the table, the disease duration affected the depressive state and fatigue, whereas relapse times or the level of neurological disturbances had no effect on the same. As for the evaluated treatment-related variables, only the length of the untreated period before starting oral PSL showed significant correlation with the evaluated insidious symptoms, but other variables (i.e., duration of oral PSL administration, present PSL daily dose, cumulative total amount of oral PSL administration, total cycles of previously performed steroid pulse therapy before the assessment, cycles of steroid pulse therapy before starting oral PSL for relapse prevention, and co-administered immunosuppressants) showed no significant correlation. The scattered plots between the treatment-related variables and the evaluated insidious symptoms are shown in Figure 1. From the figure, we can visually confirm that administration of low- to medium-dose oral PSL did not make the subsequent depressive state or fatigue deteriorate in the patients with NMOSD. Alternately, late initiation of relapse prevention therapy with immunosuppressants may.

**Table 2:** Correlation matrix between the studied symptoms and concurrent other variables in total cohort (*n* = 39).

	**Depressive state (QIDS-SR)**		**Chronic fatigue (CFS)**	
	**rho**	***p***	**rho**	***p***
**Concurrent clinical and demographic data**				
Present age	−0.006	0.974	−0.101	0.548
Disease duration [years]	+0.349	0.029	+0.419	0.009
Previous total times of relapses	+0.259	0.111	+0.206	0.215
EDSS score	+0.093	0.573	+0.033	0.846
Cerebral lesion	−0.033	0.844	−0.022	0.897
Level of gait disturbance	+0.204	0.213	+0.171	0.304
Number of eyes with legal blindness	−0.059	0.723	−0.032	0.850
**Treatment-related variables**				
Untreated period before starting oral PSL [years]	+0.384	0.016	+0.420	0.009
Duration of oral PSL [years]	+0.064	0.699	+0.085	0.614
Ratio of untreated / treated periods	+0.339	0.035	+0.353	0.030
Present oral PSL daily dose [mg/day]	−0.058	0.725	+0.029	0.861
Present oral PSL daily dose adjusted by body weight [mg/kg/day]	−0.140	0.409	−0.052	0.764
Cumulative amount of oral PSL [mg]	+0.097	0.557	+0.164	0.327
Total cycles of steroid pulse therapy before the assessment	+0.184	0.262	+0.092	0.584
Cycles of steroid pulse therapy before starting long-term oral PSL	+0.202	0.217	+0.179	0.281
Non-steroidal immunosuppressants [Yes/No]	+0.134	0.418	+0.027	0.871

*The values of Spearman's rho between the insidious symptoms and treatment-related variables are listed. As shown, none of the evaluated treatment-related variables, except for the duration of the untreated period before starting oral PSL, correlates with these insidious symptoms*.

*CFS, Chalder Fatigue Scale; EDSS, Kurtzke Expanded Disability Status Scale; PSL, prednisolone; QIDS-SR, self-reported Quick Inventory of Depressive Symptomatology*.

**Figure 2:**
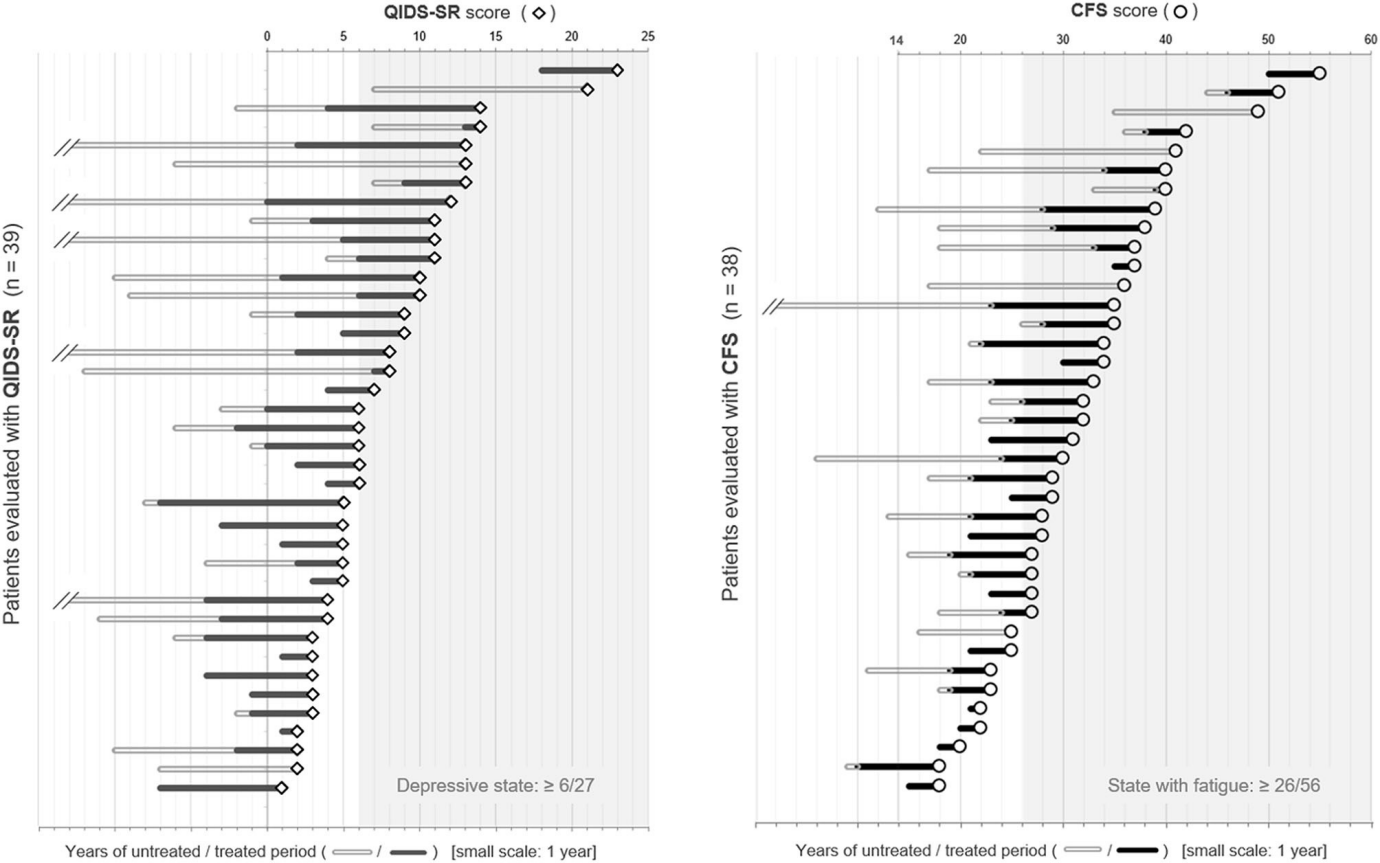
Dot plots of QIDS-SR and CFS scores with the duration of the untreated/treated periods. The patients listed were placed in order of the scores of each self-reported battery. The length of the bars on the left side of each plot represents the duration of the untreated/treated period with relapse prevention therapies in years. CFS, Chalder Fatigue Scale; QIDS-SR, self-reported Quick Inventory of Depressive Symptomatology.

To visually check the impact of the untreated period on the subsequent depressive state and fatigue level, dot plots of the scores of QIDS-SR and CFS, together with the length of untreated/treated periods with relapse prevention therapies, are shown in Figure 2. The length of disease duration and the untreated period appeared to correlate with the subsequent depressive state and fatigue level.

Since the disease duration of each patient varied, we also calculated the ratio of untreated and treated periods with relapse prevention therapies and checked for its correlation with the score of QIDS-SR or CFS, but it showed a weaker correlation with each of the insidious symptoms than the raw value of untreated years.

### Effect From the Combined Immunosuppressants

**Figure 3:**
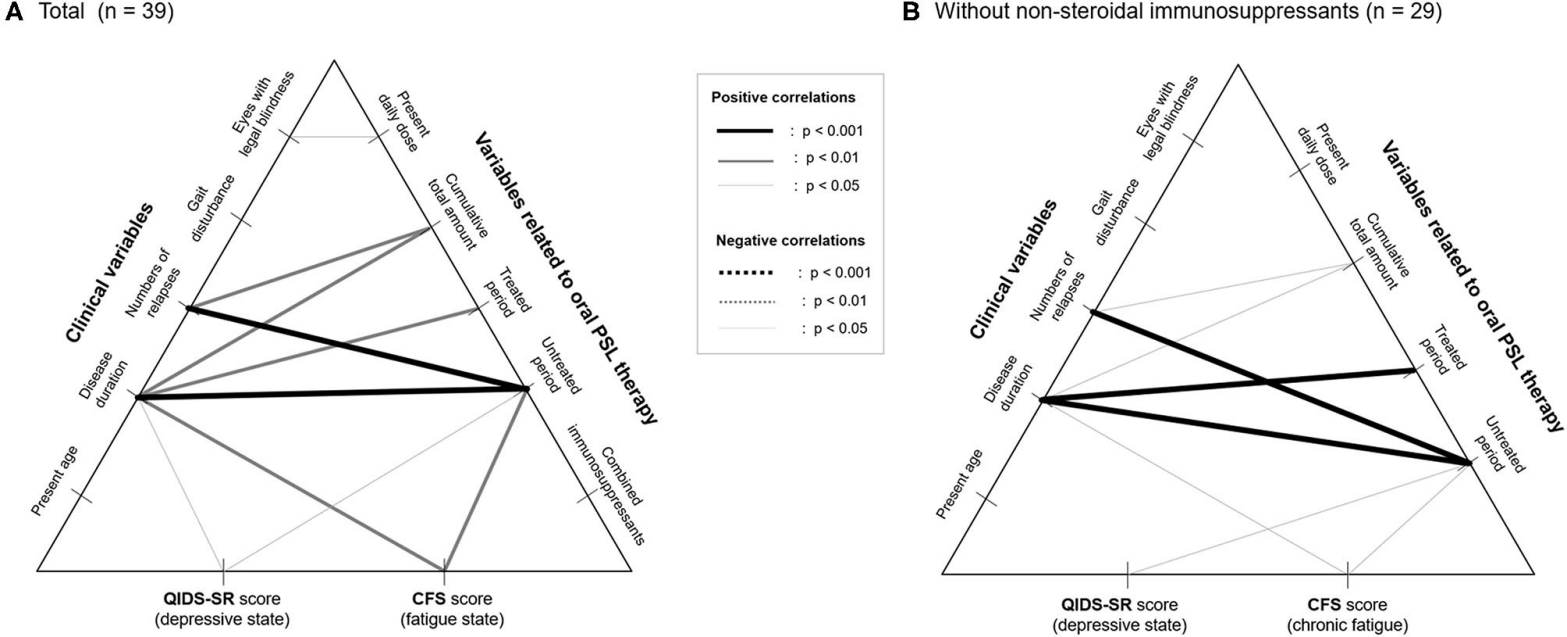
Correlation networks between clinical data, therapeutic variables, and insidious symptoms. Correlation networks in the total patients **(A)** and in those without the use of non-steroidal immunosuppressants as a potential confounder **(B)** are shown. None of the therapeutic variables, except for the duration of the untreated period with relapse prevention therapies, show a significant correlation with the evaluated insidious symptoms irrespective of the usage of nonsteroidal immunosuppressants. CFS, Chalder Fatigue Scale; PSL, prednisolone; QIDS-SR, self-reported Quick Inventory of Depressive Symptomatology.

To exclude the possible bias from the use of combined immunosuppressants, the correlations between the insidious symptoms and treatment-related variables were also calculated among the 29 patients without the use of immunosuppressants other than PSL (i.e., azathioprine, eculizumab). The results of the correlation coefficients (Table 3) show that there is no significant effect of the present PSL dosage or cumulative PSL used, on the present depressive or fatigue symptoms even after excluding the effect of the non-steroidal immunosuppressants. Moreover, the above-described negative impact of the duration of the untreated period on the subsequent insidious symptoms remained significant even after excluding the effect of co-administered immunosuppressants. Based on these results, the correlation networks between the clinical data, PSL-related variables, and evaluated insidious symptoms in the total cohort (*n* = 39) and those without non-steroidal immunosuppressants (*n* = 29) are shown in Figure 3.

**Table 3:** Correlation matrix among those without non-steroidal immunosuppressants (*n* = 29).

	**Depressive state (QIDS-SR)**		**Chronic fatigue (CFS)**	
	**rho**	***p***	**rho**	***p***
**Concurrent clinical and demographic data**				
Present age	+0.001	0.995	−0.187	0.341
Disease duration [years]	+0.340	0.072	+0.442	0.019
Previous total times of relapses	+0.333	0.078	+0.324	0.093
EDSS score	+0.068	0.725	+0.046	0.815
Cerebral lesion	−0.041	0.834	−0.007	0.971
Level of gait disturbance	+0.102	0.600	+0.063	0.749
Number of eyes with legal blindness	+0.059	0.763	+0.072	0.715
**Treatment-related variables**				
Untreated period before starting oral PSL [years]	+0.429	0.020	+0.470	0.012
Duration of oral PSL [years]	+0.084	0.666	+0.164	0.404
Ratio of untreated / treated periods	+0.384	0.040	+0.398	0.036
Present oral PSL daily dose [mg/day]	−0.008	0.968	+0.160	0.417
Present oral PSL daily dose adjusted by body weight [mg/kg/day]	−0.105	0.602	+0.120	0.558
Cumulative amount of oral PSL [mg]	+0.112	0.564	+0.275	0.157
Total cycles of steroid pulse therapy before the assessment	+0.258	0.176	+0.202	0.302
Cycles of steroid pulse therapy before starting long-term oral PSL	+0.246	0.198	+0.258	0.185

*The values of Spearman's rho between the insidious symptoms and treatment-related variables are listed. As shown, none of the evaluated treatment-related variables, except for the duration of the untreated period before starting oral PSL, correlates with these insidious symptoms*.

*CFS, Chalder Fatigue Scale; EDSS, Kurtzke Expanded Disability Status Scale; PSL, prednisolone; QIDS-SR, self-reported Quick Inventory of Depressive Symptomatology*.

## Discussion

Depressive state and chronic fatigue in patients with NMOSD are distressful conditions that occur frequently but are often overlooked or insufficiently counteracted. Shi et al. (31) demonstrated that anxiety, depressive state, neurological disability, and chronic fatigue significantly impaired the health-related QOL in NMOSD (31). In another study, Seok et al. demonstrated that a depressive state affects mental aspects, whereas chronic fatigue affects physical aspects of QOL (4). Chavarro et al. (32) showed that about 30% of patients with NMOSD experienced moderate to severe depressive states, but only 40% of these patients with depression were properly and adequately treated with antidepressants (32). These facts suggest the importance of proper assessment and therapeutic interventions needed in patients with NMOSD. However, whether the mechanism of psychiatric disturbances in patients with NMOSD and psychiatric patients is similar, remains unclear. Moreover, the effect of conventional antidepressants in treating the depressive state in NMOSD is still inconclusive (32). A previous report demonstrated that the rate of suicide attempts in patients with NMOSD with a depressed state is much higher than that in the normal population (33). Similar to the management of non-NMOSD patients with usual depression, there is a need for caution and vigilant attention toward patients with NMOSD and depression for their suicidal ideation, and immediately consult psychiatrists if the ideation seems urgent.

In this study, the present daily dose (<20 mg daily; a standard daily dose for patients with NMOSD in Japan) or cumulative total amount of oral PSL in patients with NMOSD was not significantly associated with depressive states or chronic fatigue level. To date, it is unclear whether the mid- to long-term use of low- to medium-dose oral PSL administration (i.e., <20 mg daily) may cause psychiatric disturbances or chronic fatigue. The results of this study imply that the higher levels of depression and chronic fatigue in patients with NMOSD cannot be explained by the therapeutic side-effects of steroids. Our results were comparable with several previous reports, i.e., that the risk of mental conditions with oral steroid administration is minimal with a PSL daily dose <20 mg, although these risks would dramatically increase for the administration of a daily dose of more than 40–80 mg (34, 35).

In the present study, both QIDS-SR and CFS showed weak to moderate correlation with disease duration and the untreated period before starting oral PSL. Meanwhile, these insidious symptoms showed no significant correlation with the present age or with the present level of neurological disability. These may imply a delayed impact of clinical attacks and/or unknown latent progression of glial or neural damage leading to the psychiatric condition and fatigue levels in NMOSD. A possible explanation for such delayed or latent impact is the prolonged demyelination secondary to primary astrocytic damage in NMOSD (36). Demyelination was once thought to have no association with NMOSD, but was later proved pathologically to exist in NMOSD (37). In both multiple sclerosis (MS) and NMOSD, temperature-induced conduction block in demyelinated segments is thought to cause Uhthoff's phenomenon (38, 39). Henceforth, such an activity-dependent conduction block has been proposed to contribute to fatigability in patients with MS (40, 41). Similarly, it is likely that impaired nerve conduction based on secondary demyelination in NMOSD may contribute to the delayed manifestation of depressive states and chronic fatigue in the disease. Another possible explanation for chronic fatigue is subclinical hypoxemia in patients with NMOSD, which has been proposed in a previous report (42). Chronic inflammation in the central nervous system has also been suggested to cause fatigue in patients (43, 44). These theories seem to be compatible with the observed correlation between the untreated period and subsequent depression and fatigue levels in this study. Meanwhile, among the unlikely causes of chronic fatigue in NMOSD is the serum level of L-carnitine. Generally, decreased serum L-carnitine level is considered to result in chronic fatigue, but the effect of orally administered L-carnitine on chronic fatigue in patients with MS is unsettled (5, 45). At present, there has been no report that shows a significant effect of orally administered L-carnitine on chronic fatigue in AQP4-IgG-positive patients with NMOSD.

As a limitation of this study, all the patients included were Asian, and their body weight and body surface areas were generally much lower than those in Caucasian and African American patients. Consequently, to achieve an equivalent effect of relapse suppression by oral PSL, a higher daily dose of oral PSL may be required in Caucasian and African American patients with NMOSD. Long-term administration of medium- to high-dose oral corticosteroids (i.e., above the daily dose of 7.5–10 mg) would result in side effects other than psychiatric disturbances or chronic fatigue, such as hyperglycemia, hyperlipidemia, hypertension, weight gain, infectious diseases, glaucoma, cataract, and osteoporosis (46–48). Moreover, although it is difficult to discuss the direct effect of oral glucocorticoids on the mortality rate after excluding the effect of the primary diseases, a higher daily dose of oral glucocorticoids has been suggested to be associated with an elevated risk of all-cause mortality in patients with rheumatoid arthritis (49). In the present study, we screened only for psychiatric disturbance and chronic fatigue with oral PSL, but ideally, we need to assess all the possible side effects of glucocorticoids (e.g., serum lipid levels, blood glucose level, HbA1c, blood pressure, and body mass index) to determine the tolerability of long-term oral PSL administration in patients with NMOSD. In view of these facts, if a patient with NMOSD requires an oral PSL daily dose higher than 7.5 to 10 mg as monotherapy for sufficient relapse prevention, other additional or alternative steroid-sparing relapse prevention therapies should be considered to avoid possible steroid-induced side effects.

## Conclusion

Long-term administration of low- to medium-dose oral PSL <20 mg daily would not aggravate the depressive state or fatigue level in patients with NMOSD. Alternately, early initiation of oral PSL administration for relapse prevention may alleviate the subsequent depressive and fatigue conditions. The effect of early initiation of relapse preventive therapies other than oral PSL to prevent the progression of depression and fatigue in AQP4-IgG-positive NMOSD remains to be confirmed by future studies.

## Data Availability Statement

The raw data supporting the conclusions of this article will be made available by the authors, without undue reservation.

## Ethics Statement

The studies involving human participants were reviewed and approved by Tohoku University School of Medicine. The patients/participants provided their written informed consent to participate in this study.

## Author Contributions

TA and IN contributed to study design and drafted the original manuscript. TA, TT, KF, TM, YT, SN, and IN contributed to data collection. TA, TT, TM, JF, MAb, and IN contributed to data analysis and data interpretation. KF, TI, MAo, and IN supervised all stages of the research process. All the authors were involved in the writing of the manuscript and critically revised it.

## Conflict of Interest

The authors declare that the research was conducted in the absence of any commercial or financial relationships that could be construed as a potential conflict of interest.
